# Patient-reported outcomes and complication rates after lateral maxillary sinus floor elevation: a prospective study

**DOI:** 10.1007/s00784-020-03755-x

**Published:** 2021-02-23

**Authors:** Carlo Rengo, Antonino Fiorino, Alessandro Cucchi, Antonio Nappo, Emanuele Randellini, Paolo Calamai, Marco Ferrari

**Affiliations:** 1 Department of Prosthodontics and Dental Materials, University of Siena, Viale Bracci, 53100 Siena, Italy; 2 Dentistry Unit, Catholic University of Sacred Heart, Rome, Italy; 3 Private Practice, Bologna, Italy; 4 Private Practice, Fucecchio, Italy; 5 Private Practice, Florence, Italy

**Keywords:** Patient-reported outcomes, Maxillary sinus floor augmentation, Pain, Complications

## Abstract

**Objectives:**

Oral surgery morbidity is highly variable based on patients’ characteristics and kind of surgical intervention. However, poor data are available in the literature regarding patient outcomes after oral surgery. The aim of this retrospective study was to evaluate patient-reported outcome and complication rates after maxillary sinus floor elevation.

**Materials and methods:**

Data from the records of patients undergoing maxillary sinus elevation have been collected from a private dental office. Patient-reported outcome has been assessed using a 100-mm visual analog scale to evaluate the post-operative pain (VAS_pain_) experienced in the first week following surgery and visual rating scales to evaluate discomfort level (VRS_discomfort_: 0 to 4) and willingness to repeat the same surgical procedure (VRS_willingness_: 0 to 3). Analgesics intake, swelling onset and duration, and ecchymosis have been also recorded.

**Results:**

VAS_pain_ showed moderate values in the first 2 days (< 50) post-surgery, with a tendency to progressively decrease over the next 2 days. Average assumption of painkillers was 3.93 ± 3.03. Discomfort level (VRS_discomfort_) after surgery was low (median: 1; IR: 1–0), while willingness to undergo the same surgical procedure was very high (77.63% of patients). Swelling and ecchymosis were experienced by 97.36% and 51.32% of patients, respectively, with a mean duration of 4.09 ± 1.43 and 2.21 ± 2.31 days, respectively. Membrane perforation occurred in 4 cases. Other post-operative complications were not observed.

**Conclusions:**

Maxillary sinus grafting is a safe procedure, with a low complication rate and moderate morbidity that is well tolerated by patients. Particular attention is needed in case selection, surgical planning and operator expertise.

**Clinical relevance:**

The analysis of patient-reported outcomes can be of great help in surgical planning and in providing correct and adequate treatment.

## Introduction

Oral surgery morbidity is highly variable based on patients’ characteristics and kind of surgical intervention. However, poor data are available in the literature regarding patient outcomes after oral surgery.

Actually, it is particularly difficult to objectively assess pain, since it is affected by personal control of sensations and emotions [1]. However, dental patients are more worried about pain and fear of oral surgical interventions than costs or outcomes. Therefore, management of the patient anxiety and pain is crucial in patients undergoing oral surgical procedures [2].

Generally, conventional dental implant placement causes mild to moderate pain experience [3, 4]. Implant placement in the posterior maxilla often involves additional bone augmentation procedures, when, following tooth loss, the residual alveolar ridge is reduced due to pneumatisation of the maxillary sinus or vertical resorption of the edentulous bone crest [5, 6].

Despite the introduction of xenograft or synthetic biomaterials has significantly reduced the surgical morbidity [7–10], lateral maxillary sinus floor elevation (LSFE), as first described from Boyne J and James RA [11], is still considered an invasive technique among bone regeneration procedures of the posterior maxilla [12]. Therefore other types of procedures have been suggested, including trans-crestal sinus floor elevation [13], graft-less sinus augmentation technique [14], short implants [15], tilted implants [16], pterygoid [17], or zygomatic implants [18].

Short implants are definitely associated with a minor discomfort for the patient compared to all bone augmentation procedures [19] and survival rate of implant-supported prosthesis with implant/crown ratio up to 3 is still very high [20]; thus, this approach represents a valid and predictable alternative. While some studies have shown that the success rates of short implants are even higher than those of long implants in augmented bone [21], other groups reported that the success rate is comparable with standard implants, showing less biological complications but more prosthetic complications [22].

However, recent reviews provided contradictory findings pointing out that short implants fail at an earlier stage compared to standard implants [21, 23].

Probably, these higher failure rates could be related to loading protocols or lack of adequate primary stability that could be lower for short implants [24].

Obviously, when the residual bone height is inadequate even for extra-short implants, it is necessary to resort to bone regeneration techniques [22].

Trans-crestal approach is the main alternative to LSFE and it is generally preferred by patients due to reduced morbidity, comparable to implants inserted in native bone [7], thus lower than with the lateral approach, and to the shorter period of rehabilitation [25].

The LSFE is however a predictable technique [26]. Moreover, the use of new tools such as piezoelectric devices [27] and less invasive clinical protocols [28] flap designs [29] may represent a great advantage in terms of patient benefit, pain experience, and clinical outcome. To date, there is a lot of data on the success and complication rates of this technique; however, the literature does not report the patient’s experience after this surgery [30].

Therefore, the aim of this study was to evaluate various patient-reported outcome measures (PROMs) and early complication rates in patients treated with LSFE. The primary outcome was to evaluate, through a VAS scale, the level of pain experienced by patients in the 7 days following surgery. Secondary outcomes were the evaluation of surgical discomfort, the willingness to undergo surgery again, the amount of painkillers taken, the onset and duration of swelling, and ecchymosis.

## Methods

### Study design and patient selection

The study was designed as single-center, non-randomized, prospective, observational study. All consecutive patients referred to the author for mono-lateral LSFE were enrolled and treated between February 2019 and January 2020.

Treatment planning was discussed and benefit/risk ratio was explicated to each patient that subsequently signed a written informed consent form for participation in the present study, for processing of personal data and images, and for publishing purposes, approved by local ethical committee (“Azienda Ospedaliera – Universitaria Senese” Ospedale “Le Scotte” Siena, Italy). The study obtained approval by the board of the Department of Prosthodontics and Dental Materials, University of Siena, Italy, in accordance with the Helsinki Declaration for biomedical research involving human subjects and Good Clinical Practice Guidelines (General Assembly of the World Medical Association 2014). Registration on www.clinicaltrials.gov was not necessary due to the study design, which was conducted following the STROBE guidelines [31].

The inclusion criteria were as follows: edentulism in posterior maxilla that required implant-supported rehabilitation, alveolar atrophies classes A or C, according to Chiapasco et al. [32], with residual bone height (RBH) < 6 mm, bone width ≥ 5 mm, and no vertical resorption. Exclusion criteria were: age < 18 years, any systemic disease contraindicative of surgery, un-treated periodontal disease, uncontrolled diabetes, poor horal hygiene, endodontic lesions at adjacent teeth, history of sinusitis (including present maxillary sinus infection or pathology). Smokers were informed about the increased risk of surgery and were advised to reduce/stop smoking.

### Pre-surgical procedure

After the collection of accurate medical history, a clinical and radiological evaluation (OPT and peri-apical x-rays) of each patient was performed to set the correct therapeutic plan for implant-prosthetic rehabilitation. Subsequently, patients underwent cone beam computed tomography (CBCT) x-ray examination extended to the osteo-meatal complex to evaluate bone volumes, patency of the ostium, and the presence of alterations or infections of the endosinusal mucosa. Non-surgical or surgical treatments of sinusal disease were mandatory prior to the following interventions.

Patients with periodontitis underwent cause-related periodontal therapy and were adherent to maintenance care for at least 6 months before the beginning of the study.

All patients underwent professional oral hygiene 1 week before the surgery.

Prophylactic oral premedication was adopted using amoxicillin clavulanate (1 g/8 h) or clindamycin (600 mg/12 h) in patients allergic to penicillin, starting 1 day before surgery and for 7 days after surgery. All patients rinsed their mouth for 2 min with a 0.2% chlorhexidine solution prior to surgery.

### Surgical procedure

All sinus lifts were performed by a single experienced operator under local anesthesia with articaine chloridrathe 4% and adrenaline 1:100.000, through infiltration in the buccal vestibule, at the infraorbital foramen and major palatine foramen. A full-thickness flap was elevated to expose the alveolar crest and the lateral wall of the maxillary sinus, performing a mid-crestal incision and a vertical mesial releasing incision, mesial to the last tooth before the edentulous site. The flap was raised apically to the extent to allow the positioning of a periosteal elevator or retractor, to protect soft tissues, at about 5 mm from the apical edge of the osteotomy. The triangular flap design was respected for all patients. Using a round bur mounted on a contra-angle and under sterile saline irrigation, a superficial osteotomy was performed on the lateral sinus wall until leaving a thin layer of bone subsequently removed with a round diamond tip mounted on a sonic device (Sonosurgery, TKD, Italy) under sterile saline irrigation. The coronal margin of the osteotomy was approximately 3 mm from the sinus floor.

A flat oval “elephant foot” shaped tip mounted on the sonic device was used to gently elevate the sinus membrane from the osteotomy borders. Schneider membrane elevation was continued with hand instruments until detaching the medial sinus wall, elevating the sinus floor membrane distally and mesially to allow for graft placement in the future implant receptor site, and paying particularly attention in the presence of intercalated edentulism at the level of the roots protruding in the maxillary sinus. During the surgical procedure, the possible presence of membrane perforations was evaluated through direct observation using × 2.5 magnification loupes. In case of membrane perforation, repair was attempted placing a resorbable collagen membrane (Bio-Gide**®**; Geistlich Pharma, AG, Switzerland).

Sinus was then filled with a bovine bone graft (Bio-Oss**®** Large granules 1 mm–2 mm; Geistlich Pharma, AG, Switzerland) previously mixed with sterile saline solution. The graft was initially packed tightly against the medial wall, then against the anterior and posterior compartments, and finally over the lateral sinus wall. A resorbable collagen membrane (Bio-Gide**®**; Geistlich Pharma, AG, Switzerland) was applied to cover the osteotomy and the flap was repositioned, without need of passivation or periosteal releasing incisions, and sutured with non-resorbable sutures (4.0 Polyester; Omnia, Italy).

### Post-surgical procedure

Antibiotic prophylactic was continued for 7 days after surgery and analgesics were prescribed (ibuprofen 600 mg: 1 tablet immediately after surgery and 1 tablet after 8 h). Analgesics prescription for the days following the surgery was ibuprofen 600 mg/ad libitum. Patients applied ice packs to their face for 15 min on and 15 min off for 2 h, and were asked to rinse at least twice a day with a 0.12% chlorhexidine solution and to follow a cold and soft/liquid diet for 14 days. Patients were also asked to avoid blowing their noses and a nasal decongestant spray (Tonimer Lab Hypertonic, Ist. Ganassini, Italy) was prescribed for 10 days to help maxillary sinus drainage. Sutures were removed 2 weeks after surgery during the 14-day visit.

### Radiographic evaluation

An OPT x-rays exam was performed immediately after surgery and a CBCT x-rays exam was performed 6 months after to evaluate the volume of the grafts and to plan implant placement.

#### Patient reported outcome measures and complications measure

After surgery, the patients were given a notebook to fill in to collect the outcomes. All patients were instructed by a second investigator to complete the questionnaire, clarifying any doubts regarding interpretation. An open comment section has also been included.

Patient-reported outcomes were assessed using a 10-mm visual analogue scale (VAS) to evaluate the post-operative pain experienced in the first week following surgery and visual rating scales (VRS) to evaluate discomfort level during surgery (0 = none, 1 = mild, 2 = moderate, 3 = intense, 4 = very intense) and willingness to undergo the same surgery (0 = no problem repeating the surgery, 1 = I would do it again but I would prefer to delay, 2 = I would do it again but I expect to suffer a lot, 3 = I would never do this again). These two VRS were recorded immediately after surgery.

Analgesics intake was recorded from the 2nd to the 7th post-surgical day. Onset and duration of swelling and ecchymosis (bruising, hematoma) were also evaluated for 7 days.

Finally, all complications, either observed by the operators or by the patient, were recorded and divided into: surgical complications (venous or arterial bleeding, sinusal membrane perforation, or buccal flap laceration) and healing complications (wound dehiscence, infection/fistula, or sinusitis).

Follow-up visits were scheduled 7 days after surgery, 14 days after surgery, and then monthly up to 6 months.

For data analysis, the patients were divided into different groups according to: sex, smoking habits, hypertension, residual bone height, periodontitis, and bone class.

#### Statistical methodology

An Excel data collection form and data management system was used (Microsoft Excel 2011; Windows, ver. 14.0.0; Microsoft Corp., Redmond, WA, USA). All data were entered by a single blinded operator. Prior to entry, all data were evaluated in terms of accuracy and completeness. For each continuous variable the mean, median, standard deviation (SD), interquartile range (IQR), and the maximum and minimum value were reported. Hypothesis of normality was tested with the skewness/kurtosis tests (normal distribution if *p* value > 0.05). For qualitative data frequencies, proportions and 95% confidence intervals for proportions were calculated. In bivariate analysis, proportions were compared using 2 tests. The chi-square statistic (i.e., *χ*2-statistic) were performed when no more than 20% of the cells of the contingency tables had frequencies of 5 or less and that no cells have expected frequencies less than 1 (Cochran, 1954). If any of the observed values was less than 5, then a Fisher’s exact test was performed. The comparison of means to evaluate statistically significant differences was performed by *t* test, Wilcoxon rank sum test and Wilcoxon matched–pairs signed-ranks test where necessary. Between variables where it was possible to use parametric tests, the median was not reported because both respected the assumption of normality. Where a non-parametric test was used, the median is always reported. The possible linear correlation between the different variables was investigated by applying the Pearson’s, or when the variables did not have a normal distribution or a nonlinear relationship, Spearman’s correlation test. Correlations were reported specifying the *p* value and the Pearson (*r*) or Spearman (rs) correlation coefficient. The threshold value decided for determining the statistical significance corresponds to a *p* value of 0.05 (5%). Post hoc statistical power was obtained comparing the cumulative mean of VAS pain over the 7 days of observation with a known value published in the previous literature. Calculation was performed as follows: power = Φ(−z1 − α/2 + |μ0 − μ1| × *n*^−^√/σ). The statistician was blinded and external to working group. Data analysis was performed with STATA/IC software (StataCorp LLC, College Station, TX, USA).

## Results

In total, 76 patients were treated according to the protocol procedure.

The median dose (number of vials) of anesthetic was 3 (IR, 2–4).

Osteotomy median dimensions were 10 mm (IQR, 8–12) of width and 7 mm (IQR, 6–9) of height. The surgeries had a median duration of 76 min.

All patients included for the final data analyses completed the questionnaire without omitting information. No drop-outs were reported. The characteristics and anamnestic data of overall population were collected and shown in Table 1.

**Table 1:** Patient data and characteristics (*n* = 76)

SEX (M/F)	38/38
Age (y) (range)	52 ± 10 (31–73)
Smoke	18 (23.7%)
Periodontal disease	30 (39.5%)
Systemic disease (total)	31 (40.8%)
Hypertension	12 (15.8%)
Depression	6 (7,9%)
Diabetes	2 (2.6%)
Liver disease	2 (2.6%)
Osteoporosis	2 (2.6%)
Anemia	2 (2.6%)
Concomitant medications	Anticoagulants (3); antihypertensive (2); metformina (1); antidepressive (3); eutirox (3); FANS (1); immunosuppressants (1)
Alterations of the sinusal mucosa	8 (10.5%): 7 = mild thickening of the mucosa 1 = mucous retention cyst
Bone class (A/C)	40 (52.6%)/36 (47.4%)
Residual bone height (mm)	3.7 ± 1.5
Septa	4 (5.3%)
Posterior superior alveolar artery passing through the osteotomy area	10 (13.2%)
Implants contestually	11 (14.47%)

### Patient-reported outcomes

**Fig. 1:**
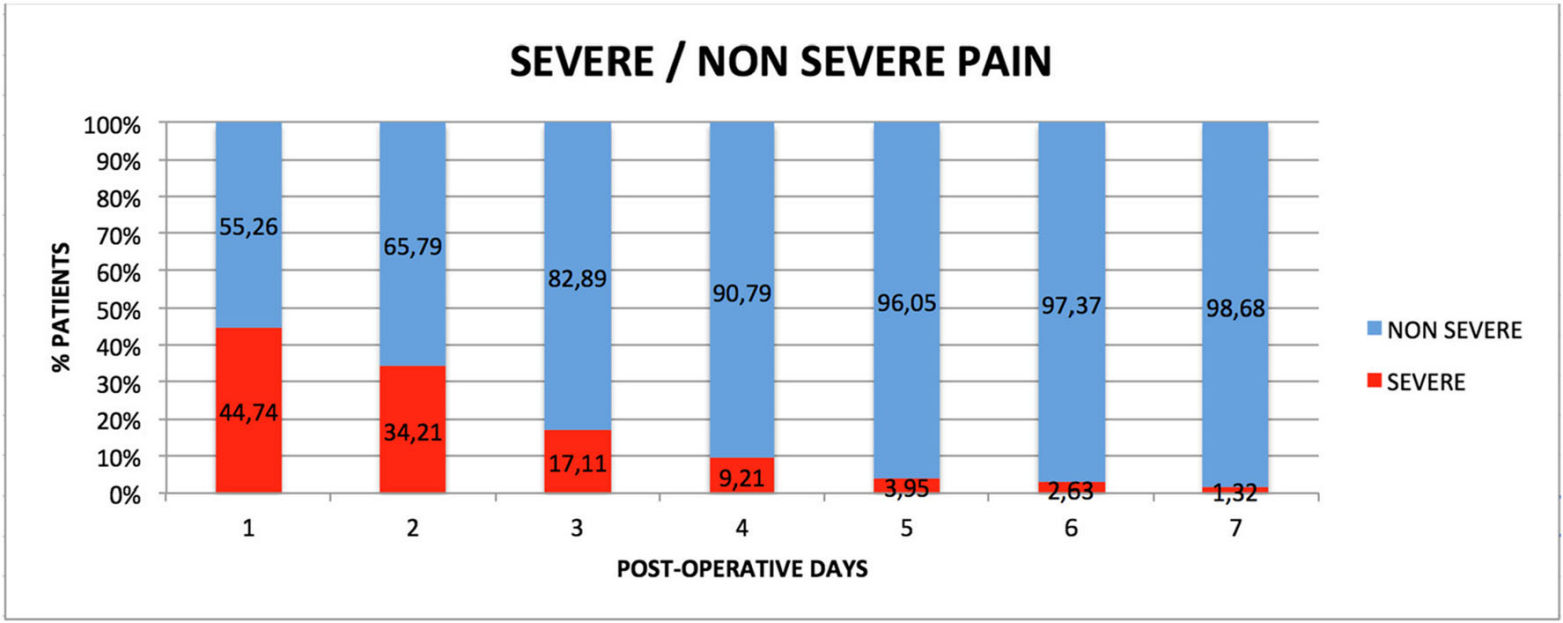
Distribution of severe pain and non-severe pain during the first post-operative week

Pain levels assessed during the week following the surgery, as total population and sorted by different groups, are reported in Table 2. The distribution of severe pain and non-severe pain is shown in Fig. 1.

**Table 2:** Average pain VAS values reported over the 7 days of observation of the entire population and stratified by groups based on sex (SEX), smokers (SMK), hypertension (HYP), residual bone height (RBH), and periodontal disease (PER). Significant intergroup differences are marked in italic. For each value, mean, standard deviation (SD), interquartile range (IQR), median, and the range of values were reported, indicating the minimum and maximum (min; max)

		Vas 1		Vas 2		Vas 3		Vas 4		Vas 5		Vas 6		Vas 7	
		Mean ± SD; (IQR)	Median (min; max)	Mean ± SD; (IQR)	Median (min; max)	Mean ± SD; (IQR)	Median (min; max)	Mean ± SD; (IQR)	Median (min; max)	Mean ± SD; (IQR)	Median (min; max)	Mean ± SD; (IQR)	Median (min; max)	Mean ± SD; (IQR)	Median (min; max)
Total population		5.07 ± 2.13; (2.5)	5 (0; 10)	4.37 ± 2.52; (3)	5(0;10)	3.27 ± 2.41 (4)	3 (0; 8)	1.94 ± 2.06 (3)	1 (0; 8)	1.10 ± 1.83 (2)	0 (0; 9.5)	0.61 ± 1.58 (0)	0 (0; 9.5)	0.36 ± 1.27 (0)	0 (0; 9.5)
Variab.	Group														
Sex	M	5.10 ± 2.25; (2.5)	5(0; 10)	4.15 ± 2.73; (3)	5(0; 10)	2.91 ± 2.53; (4)	2.5 (0; 8)	2.01 ± 2.33 (3)	1 (0; 7.5)	1.07 ± 1.95 (2)	0 (0; 9.5)	0.61 ± 1.71; (0)	0 (0; 9.5)	0.47 ± 1.63; (0)	0 (0; 9.5)
	F	5.03 ± 2.03; (1.5)	5.15 (5; 10)	4.59 ± 2.03; (2)	5(0; 9)	3.63 ± 2.27; (3.5)	4(0; 8)	1.87 ± 1.78; (1.3)	1.4(0; 8)	1.14 ± 1.72; (2)	0 (0; 6)	0.61 ± 1.48; (0)	0 (0; 6.5)	0.28 ± 0.77; (0)	0 (0; 4)
	*p* value	0.8953		0.3196		0.1483		0.6039		0.4977		0.7878		0.6350	
SMK	Y	5.91 ± 2.11; (2)	6 (1; 10)	4.92 ± 2.80; (2)	5 (0; 10)	3.96 ± 2.77; (5)	4.5 (0;8)	2.09 ± 2.18; (3.5)	1.35 (0; 6)	1.09 ± 1.65; (2)	0 (0; 5.6)	0.47 ± 1.58; (0)	0 (0;6.5)	0.06 ± 0.24; (0)	0 (0; 1)
	N	4.81 ± 2.09; (2)	5 (0; 9)	4.20 ± 2.43; (3)	5 (0; 9)	3.06 ± 2.27; (4)	3 (0; 8)	1.89 ± 2.04; (2.3)	1 (0; 8)	1.11 ± 1.89; (2)	0 (0; 9.5)	0.65 ± 1.60; (0)	0(0; 9.5)	0.46 ± 1.43; (0)	0 (0; 9.5)
	*p* value	0.0642		0.3893		0.2180		0.8033		0.9619		0.3289		01599	
HYP	Y	5.68 ± 1.85; (2.69)	2.39 (1; 7)	4.43 ± 2.86; (4.4)	5 (0; 9)	3.58 ± 2.89; (4.65)	3.75 (0; 8)	2.45 ± 2.57; (3.65)	1.85 (0; 8)	1.72 ± 2.21; (2.75)	0.75 (0; 6)	1.33 ± 2.26; (2.25)	0 (0; 6.5)	0.71 ± 1.36; (1)	0 (0; 4)
	N	4.95 ± 2.17; (2)	5 (0; 10)	4.36 ± 2.48; (3)	5 (0; 10)	3.21 ± 2.33; (4)	3 (0; 8)	1.84 ± 1.96; (2.65)	1 (0; 7.5)	0.99 ± 1.74; (2)	0 (0; 9.5)	0.47 ± 1.41; (0)	0 (0;9.5)	3 ± 125; (0)	0 (0; 9.5)
	*p* value	0.2254		0.6912		0.7684		0.5517		0.1818		0.1425		0.2476	
RBH	< 5	5.29 ± 2.25; (2)	5(0; 10)	4.68 ± 2.59; (2)	5(0;10)	3.45 ± 2.55; (4)	3.5(0; 8)	1.91 ± 2.24; (2.5)	1 (0; 7.5)	1.17 ± 2.17; (1)	0(0; 9.5)	0.29 ± 1.59; (0)	0(0;9.5)	0.42 ± 1.76; (0)	0(0;9.5)
	≥ 5	4.87 ± 2.03; (2.35)	5 (0; 9)	4.09 ± 2.46; (3.5)	5 (0; 9)	3.11 ± 2.31; (4)	3 (0; 8)	1.97 ± 1.92; (2.75)	2 (0; 8)	1.04 ± 1.47; (2)	0 (0; 6)	0.56 ± 1.13; (2.5)	0(0; 50)	0.43 ± 0.9; (0.25)	0 (0; 4)
	*p* value	0.3983		0.7825		0.0683		0.1261		0.2328		*0.0432*		0.4974	
PER	Y	5.03 ± 2.17; (4)	6 (0; 10)	4.1 ± 3.06; (6)	5 (0; 10)	3.18 ± 2.7; (5)	3 (0; 8)	1.95 ± 2.25; (3)	1 (0; 7.5)	1.03 ± 1.96; (2)	0 (0; 9.5)	0.48 ± 1.82; (0)	0 (0; 9.5)	0.42 ± 1.76; (0)	0 (0; 9.5)
	N	5.09 ± 1.67; (2)	5 (1; 9)	4.54 ± 2.12; (3)	5 (0; 9)	3.33 ± 2.24; (3.5)	3 (0; 8)	1.93 ± 1.95; (1.8)	1.4 (0; 8)	1.15 ± 1.75; (2)	0 (0; 6)	0.69 ± 1.42; (5)	0 (0; 6.5)	0.33 ± 0.82; (0)	0 (0; 4)
	*p* value	0.4641		0.7588		0.7156		0.6649		0.6825		0.1133		0.2991	
BCL	A	5.35 ± 2.38; (2.75)	5(0; 10)	4.54 ± 2.8; (3)	5 (0; 10)	3.29 ± 2.54; (4)	3 (0; 8)	1.99 ± 2.1; (2.7)	2 (0; 8)	1.28 ± 1.73; (2)	0 (0; 6)	0.5 ± 1.15; (0)	0 (0; 5)	0.32 ± 0.85; (0)	0 (0; 4)
	C	4.76 ± 1.99; (2.5)	5 (0; 7)	4.18 ± 2.20; (3)	5(0; 7)	3.25 ± 2.29; (4)	3.5 (0; 7.7)	1.88 ± 2.04; (3)	1 (0; 7.5)	0.91 ± 1.93; (1)	0 (0; 9.5)	0.73 ± 1.97; (0)	0 (0; 9.5)	0.41 ± 1.62; (0)	0 (0; 9.5)
	*p* value	0.4671		0.6201		0.9791		0.8114		03043		0.9821		0.6759

Pain level showed moderate values in the first (median, 5; IQR, 2.5; range, 0–10) and second day (median, 5; IQR, 3; range, 0–10) with a tendency to decrease over the third (median, 3; IQR, 4; range: 0–8), and fourth day (median, 1; IQR, 3; range, 0–8), showing median values of 0 from the fifth day. Total VAS average over 7 days was 2.39 ± 1.56. By comparing this value with those stated in the study of Merli et al. [33], it was possible to calculate a post hoc power equal to 100% with alpha error of 0.05%.

**Fig. 2:**
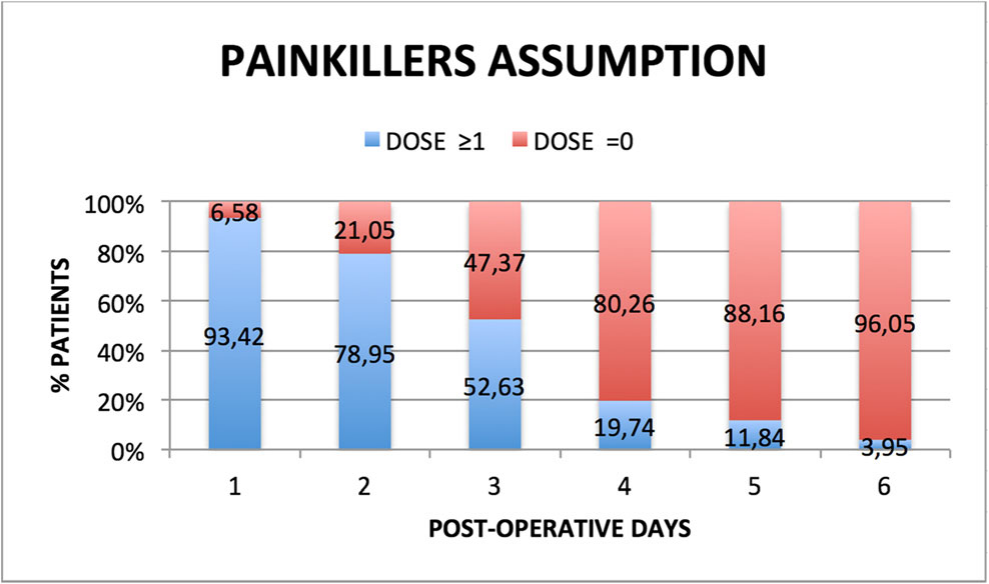
Relative percentages of patient’s painkillers assumption

**Fig. 3:**
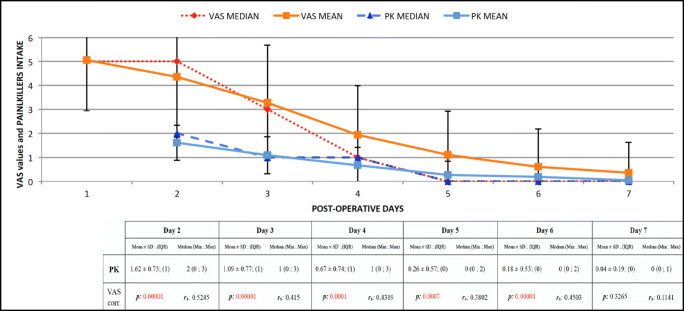
Pain VAS values and painkillers assumption during the first post-operative week

Average analgesics intake during the week following the surgery was 3.93 ± 3.03 (median, 3; IQR, 2; range, 0–14). Relative percentages of patients assuming at least one analgesic per day are showed in Fig. 2. Among the patient-related factors analyzed, painkillers assumption was significantly higher only for smokers (mean, 5.11 ± 3.56) than non-smokers (mean, 3.57 ± 2.85; *p* = 0.04) (Table 3). A strong correlation was found between VAS values and painkillers during the first 5 days (days 1–4: *p* = 0.00001; day 5: *p* = 0.0021) (Fig. 3).

**Table 3:** Painkillers assumption: the data shown are indicative of the entire population and stratified by groups based on sex (SEX), smokers (SMK), hypertension (HYP), residual bone heigh (RBH), and periodontal disease (PER). Significant intergroup differences are marked in italic and refer to the type of statistical test used. For each value, mean, standard deviation (SD), interquartile range (IQR), median, and the range of values were reported, indicating the minimum and maximum (min; max). Patient-reported painkillers assumption

		Day 2		Day 3		Day 4		Day 5		Day 6		Day 7		Total	
Variab.	Group	Mean ± SD; (IQR)	Median (min; max)	Mean ± SD; (IQR)	Median (min; max)	Mean ± SD; (IQR)	Median (min; max)	Mean ± SD; (IQR)	Median (min; max)	Mean ± SD; (IQR)	Median (min; max)	Mean ± SD; (IQR)	Median (min; max)	Mean ± SD; (IQR)	Median (min; max)
Sex	M	1.53 ± 0.80; (1)	1.5 (0; 3)	1.05 ± 0.80; (0)	1 (0; 3)	0.66 ± 0.78; (1)	0(0; 2)	0.29 ± 0.65; (0)	0 (0; 2)	0.21 ± 0.58; (0)	0 (0; 2)	0.08 ± 0.20; (0)	0 (0; 1)	3.97 ± 3.43; (2)	3 (0; 13)
	F	171 ± 0.65; (1)	2 (0; 3)	1.13 ± 0.74; (1)	1 (0; 3)	0.68 ± 0.70; (1)	1 (0; 3)	0.24 ± 0.49; (0)	0 (0; 2)	0.16 ± 0.49; (0)	0 (0; 2)	0 ± 0; (0)	0 (0; 0)	3.89 ± 2.60; (2)	3 (0; 14)
	*p* value	0.1884		0.5579		0.7367		0.9224		0.7110		0.0792		0.4768	
SMK	Y	1.78 ± 0.81; (1)	2 (0; 3)	1.39 ± 0.92; (1)	1 (0; 3)	0.83 ± 0.71; (1)	1 (0; 2)	0.44 ± 0.70; (1)	0(0; 2)	0.11 ± 0.32; (0)	0 (0; 1)	5.11 ± 3.36; (3)	4 (2; 13)		
	N	1.57 ± 0.70; (1)	2(0; 3)	1 ± 0.70; (0)	1 (0; 3)	0.62 ± 0.75; (1)	0 (0; 3)	0.21 ± 0.52; (0)	0 (0; 2)	0.14 ± 0.44; (0)	0 (0; 2)	0.02 ± 0.13; (0)	0 (0; 1)	3.57 ± 2.86; (2)	3 (0; 14)
	*p* value	0.3110		0.0868		0.2052		0.1012		0.3955		0.0759		*0.0485*	
HYP	Y	1.5 ± 0.80; (1)	1.5 (0; 3)	1 ± 0.74; (1)	1 (0; 2)	0.67 ± 0.65; (1)	1 (0; 2)	0.25 ± 0.45; (0.5)	0 (0; 1)	0.25 ± 0.62; (0)	0 (0; 2)	0.08 ± 0.29; (0)	0 (0; 1)	4.58 ± 3.96; (4)	3 (1; 14)
	N	1.64 ± 0.72; (1)	2 (0; 3)	1.11 ± 0.78; (1)	1 (0; 3)	0.67 ± 0.76; (1)	1 (0; 3)	0.26 ± 0.60; (0)	0(0; 2)	0.17 ± 0.52; (2)	0(0; 2)	0.03 ± 0.18; (0)	0 (0; 1)	3.81 ± 2.83; (1.5)	3 (0; 13)
	*p* value	0.4982		0.7256		0.8696		0.7345		0.5850		0.3983		0.9594	
RBH	< 3	1.62 ± 0.80; (1)	2 (0; 3)	1.28 ± 0.81; (1)	1 (0; 3)	0.83 ± 0.85; (1)	1(0; 3)	0.36 ± 0.68; (0.5)	0 (0; 2)	0.33 ± 0.72; (0)	0(0; 2)	0.06 ± 0.23; (0)	0 (0; 1)	4.06 ± 3.18; (1.5)	3 (0; 13)
	≥ 3	1.6 ± 0.67; (1)	2(0; 3)	0.93 ± 0.69; (1)	1 (0; 2)	0.53 ± 0.60; (1)	0 (0; 2)	0.18 ± 0.45; (0)	0 (0; 2)	0.05 ± 0.22; (0)	0 (0; 1)	0.03 ± 0.16; (0)	0 (0; 1)	3.82 ± 2.92; (2)	3 (0; 14)
	*p* value	0.7825		0.0683		0.1261		0.2328		*0.0432*		0.4974		0.8233	
PER	Y	1.4 ± 0.81; (1)	1 (0; 3)	1 ± 0.87; (1)	1 (0; 3)	0.6 ± 0.77; (1)	0 (0; 2)	0.33 ± 0.71; (0)	0(0; 2)	0.2 ± 0.61; (0)	0 (0; 2)	0.07 ± 0.25; (0)	0 (0; 1)	3.53 ± 3.28; (2)	3 (0; 13)
	N	1.76 ± 0.64; (1)	2(0; 3)	1.15 ± 0.70; (1)	1 (0; 3)	0.72 ± 0.72; (1)	1(0; 3)	0.21 ± 0.47; (0)	0 (0; 2)	0.17 ± 0.49; (0)	0 (0; 2)	0.02 ± 0.15; (0)	0 (0; 1)	4.20 ± 2.86; (2)	3 (0;14)
	*p* value	*0.0409*		0.2817		0.3915		0.7826		0.7762		0.3287		0.1611	
BCL	A	1.62 ± 0.74; (1)	2(0;3)	1.18 ± 0.78; (0.5)	1(0; 3)	0.63 ± 0.77; (1)	0 (0; 3)	0.28 ± 0.6; (0)	0(0; 2)	0.2 ± 0.56; (0)	0 (0; 2)	0.08 ± 0.27; (0)	0(0; 1)	3.9 ± 3.3; (2)	3 (0; 14)
	C	1.61 ± 0.73; (1)	2(0; 3)	1 ± 0.76; (2)	1 (0; 2)	0.72 ± 0.7; (1)	1 (0; 2)	0.25 ± 0.55; (0)	0 (0; 2)	0.17 ± 0.51; (0)	0(0; 2)	0 ± 0; (0)	0 (0; 0)	9.97 ± 2.74; (2)	4 (0; 112)
	*p* value	0.9039		0.4453		0.4210		0.9223		0.8383		0.0958		0.3412

Discomfort level was low (median, 1; IQR, 1; mean, 0.79 ± 1.01; range, 0–4) (Table 4). Patients who experienced the highest discomfort level during surgery (4 on a 0–4 scale) reported statistically significantly higher VAS values for the first 3 days (mean, 96.67 ± 5.77; median, 100), and on the 7th day together with patients who referred a discomfort level of 3 (day 1: *p* = 0.0473; day 2: *p* = 0.0481; day 3: *p* = 0.0259; day 7: *p* = 0.0401) (Table 5).

**Table 4:** Patient-reported discomfort level. Table 4 Discomfort: patient discomfort levels are reported as probability distributions and mean difference in the different groups. The data shown are indicative of the entire population and stratified by groups based on sex (SEX), smokers (SMK), hypertension (HYP), residual bone heigh (RBH), and periodontal disease (PER). Significant intergroup differences are marked in italic and refer to the type of statistical test used. For each value, mean, standard deviation (SD), interquartile range (IQR), median, and the range of values were reported, indicating the minimum and maximum (min; max)

		0	1	2	3	4	Intergroup difference (Mann-Whitney)
		N° of subjects (relative percentage)					Mean ± SD; (IQR)–median (min; max)
SUB	Total	37 (48.68)	26 (34.21)	8 (10.53)	2 (2.63)	3 (3.95)	0.79 ± 1.01; (1)–1 (0; 4)
SEX	M	16(42.1)	16 (42.11)	3 (7.89)	1 (2.63)	2 (5.26)	0.87 ± 1.04; (1)–1 (0; 4)
	F	21(55.26)	10 (26.32)	5 (13.16)	1 (2.63)	1 (2.63)	0.71 ± 0.98; (1)–0 (0; 4)
	Fisher’s exact test	**0.5720**					**0.4026**
SMK	Y	9 (50)	7 (38.89)	0 (0)	0 (0)	2 (11.11)	0.83 ± 1.24; (1)–0.5 (0; 4)
	N	28 (48.28)	19 (32.76)	8 (6.1)	2 (3.45)	1 (1.72)	0.78 ± 0.94; (1)–1 (0; 4)
	Fisher’s exact test	**0.3650**					**0.8056**
HYP	Y	6 (50.00)	3 (25.00)	1 (8.33)	1 (8.33)	1 (8.33)	1 ± 1.37; (1.5)–0.5 (0; 4)
	N	31 (48.44)	23 (35.94)	7 (10.94)	1 (1.56)	2 (3.12)	0.75 ± 0.94; (1)–1 (0; 4)
	Fisher’s exact test	**0.3650**					**0.8056**
RBH	< 3	19 (52.78)	10 (27.78)	5 (13.89)	0 (0)	2 (5.56)	0.78 ± 1.07; (1)–0(0; 4)
	> 3	18 (45.00)	16 (40.00)	3 (7.50)	2 (5.00)	1 (2.50)	0.8 ± 0.97; (1)–1 (0; 4)
	Fisher’s exact test	**0.4650**					**0.718**
PER	Y	11 (36.67)	13 (43.33)	4 (13.33)	0 (0)	2 (6.67)	0.97 ± 1.07; (1)–1(0; 4)
	N	26 (56.52)	13 (28.26)	4 (8.70)	2 (4.35)	1 (2.17)	0.67 ± 0.97; (1)–0(0; 4)
	Fisher’s exact test	**0.2540**					**0.1310**
BCL	A	21 (52.5)	13 (32.5)	1 (2.5)	2 (5)	3 (7.5)	0.83 ± 0.12; (1)–0 (0; 4)
	C	16 (44.44)	13 (36.11)	7 (19.44)	0 (0)	0 (0)	0.07 ± 0.08; (1)–1 (0; 2)
	Fisher’s exact test	*0.0320*					**0.5945**

**Table 5:** Relationship between VAS pain values and willingness/discomfort level. Table 5 Relationship between VAS pain values and willingness/discomfort levels: pain VAS values at different levels of discomfort (D) and willingness (W) in the 7 days of observation. Significant intergroup differences were marked in italic and were calculates by Kruskal-Wallis H test. For each level of D and W, the number of patients with correspondence of the VAS value indicated as median, interquartile range (IQR), and range (min; max) was reported

			VAS 1		VAS 2		VAS 3		VAS 4		VAS 5		VAS 6		VAS 7	
	Level	N°	Median–IQR (min; max)		Median–IQR (min; max)		Median–IQR (min; max)		Median–IQR (min; max)		Median–IQR (min; max)		Median–IQR (min; max)		Median–IQR (min; max)	
D	0	37	5	1 (0; 8)	5	2 (0; 8)	3	4 (0; 8)	1	2 (0; 6)	0	1 (1; 5.6)	0	0 (0; 6.5)	0	0 (0; 1)
	1	26	5	3.5 (0; 8)	5	3 (0; 7)	3.5	3 (0; 7)	1.5	3 (0; 6)	0	2 (0; 5)	0	2 (0; 3)	0	0 (0; 2)
	2	8	4	5 (5; 7)	2.5	5.6 (0; 65)	1.8	3.3 (0; 5)	1.6	3.5 (0; 7.5)	0.6	3 (0;9.5)	0.6	2.5 (0; 9.5)	0	1.6 (0; 9.5)
	3	2	4.3	4.5 (2; 6.5)	3.3	6.5 (0; 65)	2.3	4.5 (0; 4.5)	2.3	4.5 (0; 4.5)	1.8	3.5 (0; 3.5)	1.3	2.5 (0; 2.5)	1.3	2.5 (0; 2.5)
	4	3	10	1 (9; 10)	10	1 (9; 10)	8	0 (8; 8)	6	20 (6; 8)	3	3 (3; 6)	0	5 (0; 5)	0	4 (0; 4)
	*p* value		*0.0425*		*0.0481*		*0.0259*		0.0518		0.0585		0.0762		*0.0401*	
W	0	59	5	2 (0; 8)	5	3 (0; 8)	3	4 (0; 7.7)	1	2 (0; 7.5)	0	1 (0; 9.5)	0	0 (0; 9.5)	0	0( 0; 9.5)
	1	12	6	4.5 (0.5; 8)	5.5	4.5 (0; 8)	4.5	4.5 (0; 8)	2.5	4; (0; 6)	1	3.5 (0; 5)	0	2 (0; 3)	0	0 (0; 1)
	2	1	3	0 (3; 3)	3	0 (3; 3)	1.5	0 (1.5; 1.5)	0	0 (0; 0)	0	0 (0; 0)	0	0 (0; 0)	0	0 (0; 0)
	3	4	9.5	30 (5; 10)	9.5	5.1 (0.8; 10)	8	3.6 (0.9; 8)	6	3.5 (1.1; 8)	3	2.4 (1.2; 6)	0.6	3.1 (0; 5)	0.6	2.6 (0; 4)
	*p* value		*0.0450*		0.189		0.1208		*0.0241*		*0.0104*		0.2101		0.2236	

Willingness to undergo the same surgery was very high (77.63% of patients; median, 0; range, 0–3; IQR, 0; mean, 0.34 ± 0.75) (Table 6). Patients with lowest willingness (3 on a 0 to 3 scale) reported mean VAS values statistically significantly higher on days 1 (*p* = 0.0450), 4 (*p* = 0.0241), and 5 (*p* = 0.0104) (Table 5).

**Table 6:** Patient-reported willingness level. Table 6 Willingness: patiens willingness levels are reported as probability distributions and mean differences in the different groups. The data shown are indicative of the entire population and stratified by groups based on sex (SEX), smokers (SMK), hypertension (HYP), residual bone heigh (RBH), and periodontal disease (PER).Significant intergroup differences are marked in red and refer to the type of statistical test used. For each call, mean, standard deviation (SD), interquartile range (IQR), median, and the range of values were reported, indicating the minimum and maximum (min; max)

		0	1	2	3	Intergroup different (Mann-Whitney)
		N° of subjects (relative percentage)				Mean ± SD; (IQR)–median (min; max)
SUB	Total	59 (77.63)	12 (15.79)	1 (1.32)	4 (5.96)	0.34 ± 0.75; (0)–0(0; 3)
SEX	M	33 (86.84)	3 (7.89)	0 (0)	2 (5.26)	0.24 ± 0.71; (0)–0(0; 3)
	F	26 (68.42)	9 (23.68)	1 (2.63)	2 (5.26)	0.45 ± 0.80; (1)-0(0; 3)
	Fisher’s exact test	0.1960				0.0684
SMK	Y	14 (77.78)	2 (11.11)	0 (0)	2 (11.11)	0.44 ± 0.98; (0)–0(0; 3)
	N	45 (77.69)	10 (17.24)	1 (1.72)	2 (3.45)	0.31 ± 0.68; (0)–0(0; 3)
	Fisher’s exact test	0.5360				0.9063
HYP	Y	11 (91.67)	0 (0)	0 (0)	1 (8.33)	0.25 ± 0.87; (0)–0(0; 3)
	N	48 (75.00)	12 (18.75)	1 (1.56)	3 (4.69)	0.36 ± 0.74; (0.5)–0(0; 3)
	Fisher’s exact test	0.4020				0.2597
RBH	< 3	27 (75.00)	7 (19.44)	0 (0)	2 (5.56)	0.36 ± 0.76; (0.5)–0(0; 3)
	> 3	32 (80.00)	5 (12.50)	1 (2.50)	2 (5.00)	0.33 ± 0.76; (0)–0(0; 4)
	Fisher’s exact test	0.4650				0.6520
PER	Y	23 (76.67)	5 (16.67)	0 (0)	2 (6.67)	0.37 ± 0.81; (0)–0(0; 3)
	N	36 (78.26)	7 (15.22)	1 (2.17)	2 (4.35)	0.33 ± 0.73; (0)–0(0; 0)
	Fisher’s exact test	1.0000				0.8665
BCL	A	33 (82.5)	4 (10)	0 (0)	3 (7.5)	0.32 ± 0.83; (0)–0(0; 3)
	C	26 (72.22)	8 (22.22)	1 (2.78)	1 (2.78)	0.36 ± 0.68; (1)–0(0; 3)
	Fisher’s exact test	0.2520				0.3558

**Fig. 4:**
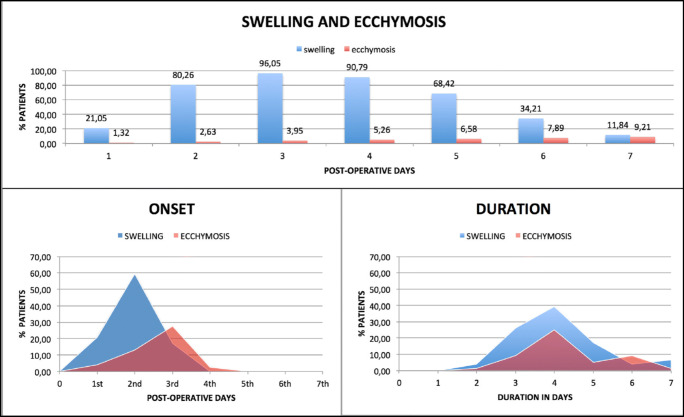
Relative percentages of patients who experienced swelling and ecchymosis in the first post-operative days. Onset and duration of swelling and ecchymosis

Swelling was experienced by 97.36% of patients with an onset observed mostly (59.21%) on the second day and never beyond the third day. Mean duration was of 4.09 ± 1.43 days. Those who had persistent swelling beyond day 5 also had significantly more pain on the 6th day (*p* = 0.0270) than the others patients. Ecchymosis was experienced by 51.32% of patients with a mean duration of 2.21 ± 2.31 days with an onset observed mostly (44.74%) within the third day (Fig. 4).

Patients who developed ecchymosis in the first 2 days (67.56%) had statistically significantly less pain on days 6 and 7 (*p* < 0.05).

Sex, age, periodontal disease, hypertension, bone class, and RBH did not influence the analgesics intake (Table 3). VAS values, discomfort level, and willingness were not influenced by any of the patient-related factors except for bone class C patients who experienced a statistically significantly higher discomfort during surgery (*p* = 0.032) (Table 4).

#### Complications and success rates

Surgical complications included 4 membrane perforations (5.26%), while healing complications included 2 wounds healed slightly by secondary intention (2.63%), giving a total complication rate of 7.89%.

In all cases of membrane perforation, the post-surgical OPT x-rays exam performed showed the graft well contained under the sinus membrane, as well as the CBCT x-rays exam performed at 6 months. During the follow-up visits, these patients did not show any signs or symptoms of sinusitis or infection, which were accurately investigated. Patients with secondary intention wound healing were visited weekly until complete healing. In the meanwhile, they were asked to continue with cold and soft/liquid diet and rinse twice a day with a 0.12% chlorhexidine solution until complete healing which occurred in all cases within the third and fourth week after surgery.

All patients showed in CBCT an adequate bone volume after 6 months allowing to place the planned implants in the correct position and axis. No complications or adverse reactions were observed during implant surgery. At re-entry surgery, all implants were successfully osseointegrated and successively loaded, giving an osseointegration rate of 100%.

## Discussion

The main objective of this study was to evaluate data about PROMs following LSFE and to investigate association with influencing factors. PROMs are useful tools that help the clinician to obtain information on the patient’s experience and on certain aspects following interventions such as symptoms, condition, and overall quality of life. These data may concur to the decision-making process and provide the patient adequate information about risk factors, post-operative experience, and recovery after surgery, definitely providing an optimal and adequate oral health treatment.

LSFE is considered a quite invasive technique. Discomfort during surgery is generally quite mild, while post-surgical pain, swelling, and ecchymosis are the most common observed signs and symptoms. Nevertheless, to date, only little piece of literature has explored this field [8, 30, 34, 35].

In the present study, post-surgical pain was evaluated by the patients through a 0–10 points VAS during the week following LSFE. Secondary outcomes were: discomfort experienced during surgery, willingness to undergo the same surgery, onset and duration of swelling and ecchymosis, analgesics intake, and complications rate.

The recent systematic review by Younes et al. [30] analyzed 11 studies about PROMs after LSFE. Unfortunately, due to the high heterogeneity in study design (1 cohort study; 1 retrospective case series; 2 prospective case series; 7 randomized controlled trials), graft materials and evaluation of outcome variables, only a descriptive data analysis was provided. The review included studies in which either uni-lateral, or bi-lateral or both sinus lift procedures were performed. The number of subjects included in most of the studies was generally very low: 9 studies enrolled less than 40 patients. Data regarding pain was reported in 8 of the 11 studies, using a 0–10 [36, 37] or 0–100 VAS [38, 39], or 3- to 5-point scales [33, 40–42]. Pain was evaluated for 7 days in all studies, with the exception of Ozturan et al. (4 days) [39], Deppe et al. [33], (14 days), Nickenig et al. (day of the surgery, 1st and 7th post-surgical day) [36], and Farina et al.(first week and 14th day) [34]. In the study of Pieri et al. [42], PROMs were evaluated only after the reconstructive procedure and implant placement. The inclusion of this article in a review analyzing PROMs after LSFE is quite questionable. Edema was documented in only 4 studies [38, 40–42]. Among other outcome measures, some studies also reported on the ability to eat and work, and phonetics [38, 40, 41], bleeding, sleeping [38, 40], and the breathing capability [38]. Mardinger et al. used two combined and adjusted Health-Related Quality Of Life (HRQOL) questionnaires for all evaluations [41]. An Oral Health Impact Profile-14 (OHIP-14) was used in 1 study to assess patient satisfaction [39].

Our findings indicate that post-operative pain has moderate values in the first 2 days with a gradual decline until the 7th day. From the 5th day, the median was 0; however, on the 7th day, still 15.78% of patients suffered pain, similarly to what observed by Mardinger et al. (11.8%) [41], suggesting to extend the reporting period in future studies. These findings are in line with previous studies that showed a pain peak on the day of surgery or first post-operative day followed by a gradual decrease in the following [30, 36–41] Conversely, in the study of Merli et al. [37], pain VAS values reported, after a one-stage LSFE procedure with conscious sedation, were very low (lower than 2 on a 0–10 scale), but with a different and conflicting trend (day 1: 1.5 ± 1.7; day 2: 1.0 ± 1.3; day 3: 0.9 ± 1.5; day 4: 1.2 ± 2.0; day 5: 1.3 ± 2.1; day 6: 1.2 ± 1.9). Similarly in a study comparing lateral or trans-crestal approach, both procedures resulted in very low pain VAS values, lower than 25 on a 0–100 scale, with higher values for the trans-crestal approach [34].

In the present article, beyond mean and median pain VAS values, also the prevalence of severe pain (VAS value > 50) was provided, observing that severe pain until the 7th day was reported by one patient only. This information is clinically relevant, but to date, only a study provided it, showing that 90% of patients experienced pain but only 8% had severe pain [33].

For comparison, this surgical intervention has shown to determine lower morbidity than the mandibular third molar extraction, except for swelling [41]. It should be taken into account that third molar extraction is usually performed in younger patients [43], with different psychological attitude and biological conditions that may influence pain experience and recovery time.

In the present study, no differences in pain based on age or gender were observed, in agreement with most of the articles in literature except for a study, in which female patients experienced more pain [41]. It should be considered that there might be other factors that can influence pain, which do not depend on the patient, but on the study design and surgical protocol.

In this study, painkillers assumption (ibuprofen 600 mg) in the days following surgery was self-administered by patients. A similar protocol was adopted in some studies with changes in dosage (600 mg or 400 mg) [34, 36, 38] or medication (paracetamol 500 mg) [39]. In the study of Farina et al., dexamethasone i.m. was given in addition to ibuprofen 600 mg [34]. In other studies, painkillers were prescribed (ibuprofen or paracetamol) for 3 to 4 days after surgery regardless of pain [37, 40], or in combination with conscious sedation and intra-venous administration of tramadol 100 mg, ketorolac 30 mg, and betamethasone sodium phosphate 4 mg [37]. Other studies did not clearly describe the drug protocol [44] [41].

The morbidity of this surgical procedure is lower than other GBR ones, and this may probably depend on the reduced surgical time, and on the management of soft tissues, without periosteal releasing incisions, and with closure by primary intention.

Regarding surgical time, it could significantly influence the levels of pain and discomfort during surgery [45]. It depends on several variables such as operator skills, the occurrence of intra-surgical complications, the use of piezoelectric devices or rotary instruments [34, 40], or autologous bone grafting [37].

Also the size of the osteotomy could affect pain levels and surgical time in a reciprocal way. A smaller osteotomy initially requires less time, but however it can lead to greater difficulties in lifting the sinus membrane, with consequent increase in surgical time and possible intra-surgical complications. In the present study, we adopted a relatively small osteotomy dimension, similarly to Farina et al. [34].

The flap design should be strongly taken into account. It was observed that flap elevation made with two vertical incisions (trapezoidal flap design) gave more pain and swelling than one incision (triangular flap design) [29]. Among the studies considered, the flap design was very [34, 37, 38] or not clearly specified [39–41].

With regard to the discomfort during surgery, in the present study, it was very low. Patients who had major surgical discomfort had also experienced more pain. This remarks how surgical experience affects the perception of pain and vice versa. Post-operative discomfort can be effectively alleviated or even eliminated through various conscious or moderate sedation procedures [37, 46, 47]. The evaluation of discomfort during surgery is clinically very relevant, because it helps the clinician to communicate to the patient what really awaits him during the surgery.

As well as discomfort, the willingness to undergo the same surgery again was also very high (77.63% of patients). We found only a study reporting on it, with a definitely lower outcome (50%) [34]. A certain relationship between willingness and pain can be hypothesized, since patients from the present study, who had a lot of pain the day after the surgery and on days 5 and 7, expressed low willingness to undergo the same surgery.

Regrettably, swelling was the most evident sign (97.36% of patients). We must point out that this parameter is difficult to assess in a quantitative or qualitative way, and generally, it is assessed subjectively by the patient, as well as ecchymosis. We found an article that attempted a quantitative evaluation through an optical 3-D imaging analysis for facial volumetric changes [36]. In an another study, the distance between the gonion and the external canthus of the eye was measured [39]. Scarano et al. used thermal infrared imaging [29].

Our observations regarding swelling were comparable to previous researches. Del Fabbro et al. observed the highest level of swelling on the 1st post-surgical day, with a gradual decline until 7th day. Delilbasi et al., observed highest values at 36 h, with a gradual decline thereafter. Mardinger et al. reported a median value of 5 (on a 0–5 VRS) on the 2nd day after surgery, with a decline until the 7th day on which 21% of patients still reported swelling. Farina et al. observed a peak on days 1 and 2 with a gradual decline up to the 7th day. Scarano et al. observed a mean value of 3.27 ± 0.59 (on a 0–5 VRS) on the 2nd day and reduced values until the 6th day, reaching no swelling at 14 days.

We have found correlation between swelling and pain. It should be considered that anti-edema drugs, such as corticosteroids, have not been used, as well as painkillers were prescribed ad libitum, to prevent post-surgical medications from being confounding factors. For this reason, the comparison of our findings with other studies in the literature is difficult and must be interpreted taking into account that the post-surgical instructions are very variable or not clearly defined [12, 33]. In the study of Schwartz-Arad et al., dexamethasone was prescribed, but swelling and hematoma were very frequent [48]. Pieri et al. prescribed betamethasone in addition to ibuprofen and reported a low prevalence of edema [42]. No corticosteroids were prescribed by Scarano et al., but Ibuprofen was prescribed to all patients for 3 days [29]. Edema was reported by only 1.6% of patients by Moreno et al. including dexamethasone in the anesthetic induction and oral non-steroidal anti-inflammatory drugs and analgesics were prescribed to all patients for 8 days [9].

Ecchymosis, which is not often reported in literature, was found in only 51.32% of the patients in our study. Those who had early bruising had less pain afterwards. Similar results were observed by Merdinger et al. (56%) [41]. In a study in which plasma rich in growth factors was used, ecchymosis was experienced by 60% of patients [38]. From our experience and from the open comments left by the patients, bruising does not particularly bothers the patient. If properly advised about this possibility, patients accept it well.

Regarding complications after LSFE, the most frequent is membrane perforation, followed by ostium obstruction and infections [48]. Membrane perforation has some clinical implications such as increased susceptibility to infections or an inadequately contained graft and may influence the implant survival rate [49]. In the present study, we did not find severe healing complications and, compared to other studies [9, 27, 28, 40, 48–53], a very low percentage of surgical complications: only 4 membrane perforations (5.2%). It should be emphasized that in this study, patient selection, accurate diagnosis, and careful therapeutic planning were scrupulously aspired. The use of a sonic device is undoubtedly another reason that could justify these findings, as reported in a recent meta-analysis [27]. Also the experience of the surgeon is crucial.

The major limit of this study was intrinsic to the aim itself, that is, measuring something like pain and discomfort, which are subjective variables, difficult to objectively quantify, as well as swelling. Another limitation is the design of the study, as it lacks a control group and therefore it was not possible to apply a randomization.

Not having adopted standard measurement systems such as OHIP-14 or OHRQOL, limits the comparison of the findings obtained with some present in the literature. We believe that these systems were not adequate for the specific topic covered, but too generic and unable to focus on the type of intervention performed. However, we have adopted a large number of measurements, hard to find in most of the studies at the moment. Data collection was very extensive and through an accurate statistical analysis we were able to show very detailed findings. Patient selection and post-surgical medications were performed in order to reduce as much as possible the presence of biases.

Future studies should have a larger sample size, a randomized controlled study design and analyze more variables, possibly more objectively, and for a longer observation period. Modified or customized OHIP- or OHRQOL questionnaire are auspicable.

Finally, since pain experienced by the patient can be connected or depend on his anxiety, future studies should contemplate anxiety control protocols that could lead to an improvement in the patient’s experience.

This study confirmed that LSFE is a safe and predictable procedure because of moderate patient morbidity and very low complication rates. High level of willingness to undergo the same surgery should encourage clinicians to choose LSFE for bone augmentation in posterior maxilla.
